# X-ray Crystallography, DFT Calculations and Molecular Docking of Indole-Arylpiperazine Derivatives as α_1A_-Adrenoceptor Antagonists

**DOI:** 10.3390/molecules201119651

**Published:** 2015-10-30

**Authors:** Wei Xu, Jun-Jun Huang, Bin-Hao Shao, Xing-Jie Xu, Ren-Wang Jiang, Mu Yuan

**Affiliations:** 1 Pharmaceutical Research Center, Guangzhou Medical University, 195# Dongfengxi Road, Guangzhou 510182, China; xwnail2003@163.com (W.X.); huangjunjun1985@sina.com (J.-J.H.); sbh1539@sina.cn (B.-H.S.); xuxingjie2015@163.com (X.-J.X.); 2 School of Pharmaceutical Sciences, Jinan University, Guangzhou 510632, China; rwjiang2008@126.com

**Keywords:** indole-arylpiperazine, single-crystal, DFT calculations, molecular docking, α_1A_-adrenoceptor

## Abstract

Indole-arylpiperazine derivatives have exhibited good selectivity for the α_1A_-adrenoceptor, but the structure-activity-binding mechanism relationship remains unclear. In the current study, three compounds (**1**, **2** and **3**) were investigated through single-crystal X-ray diffraction analysis, density functional theory (DFT) calculations and molecular docking using a homology model of the α_1A_ receptor. Compounds **1** and **3** form H-bonds networks to stabilize their three-dimensional structures, while C–H···π interactions play a significant role in the packing of **2**. Based on DFT-optimized conformations, the HOMO-LUMO energy gaps and molecular electrostatic potential (MEP) were theoretically calculated at the B3LYP/6-311G (d, p) level of theory. Chemical reactivity increases in the order of **3** < **2** < **1**, and the maximum positive region of the MEP maps is mainly localized over the NH group. The binding mechanisms of ligand-α_1A_-adrenoceptor complexes were illustrated by molecular docking. Binding to Gln177 of the second extracellular loop region via hydrogen bonds is likely to be essential for α_1A-_selective antagonists. The present work sheds light on the studies of structure-activity-binding mechanism and aids in the design of α_1A_ antagonists with high selectivity.

## 1. Introduction

Benign prostatic hyperplasia (BPH) is a progressive condition characterized by a nodular enlargement of the prostate resulting eventually in obstruction of the urethra [1]. BPH affects as many as 60% of men over the age of 60 and the number of patients is rising worldwide as a result of the aging male population [2]. The disease is characterized by obstructive and irritating symptoms and significantly compromises the quality of life of patients. Estimates of annual pharmaceutical sales of BPH therapies range from $US 3 to 10 billion, and emerging contenders to current therapies is focusing on drug targets which are able to relax prostatic smooth muscle in a similar way to the α_1_-adrenoceptor (α_1A_-, α_1B_- and α_1D_-AR) antagonists, as this appears to be the most effective mechanism of action [3]. The α_1A_-AR subtype is the predominant receptor causing contraction of the prostate smooth muscle and is thought to result in BPH. A potent and α_1A_-selective adrenoceptor antagonist can be an attractive drug candidate for treatment of BPH with fewer undesirable side effects that may be associated with α_1B_ subtype [4].

**Figure 1:**
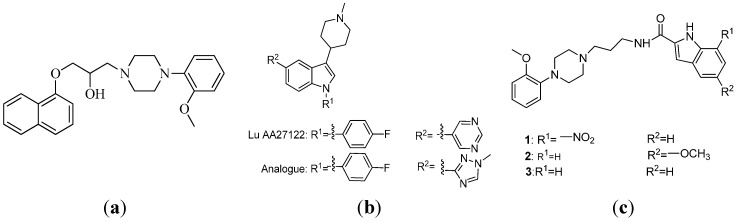
Chemical structures of naftopidil (**a**); Lu AA27122 and its analogue (**b**); and the title compounds **1**, **2** and **3** (**c**).

Arylpiperazine derivatives constitute a class of α_1_-AR antagonists [5,6,7]. Naftopidil (Figure 1a) is a clinical drug for the treatment of BPH, and this compound reflects the successful application of the arylpiperazine skeleton [8]. Several types of these derivatives exhibit high affinity for α_1_-AR *in vitro*; these compounds include pyridazinone-arylpiperazines [9], arylpiperazine derivatives bearing a flavone nucleus [10], quinazolinone-arylpiperazine [11] and 2-pyridone derivatives of arylpiperazine [12]. Aroyl piperazines can effectively reduce the prostate weights of mature rats *in vivo* [1]. Our research group also validated that naftopidil-related derivatives are potential multipotent agents that exhibit α_1_-AR blocking activity and antiproliferative activity against human prostate cancer [13]. Thus, considerable attention has been given to the arylpiperazine scaffold in the field of drug discovery because of its unique biological function.

Agents containing the indole moiety exhibited various biological activities including enzyme inhibition [14,15], tubulin polymerization inhibition [16] and anticancer activity in human HCT116 colon and HepG2 liver carcinoma cells [17]. In particular, pyrimido[5,4-*b*]indoles were reported to act as the selective α_1_-AR subtypes antagonists [18,19]. Lu AA27122 and its analog (Figure 1b) were identified as high affinity α_1A_-adrenoceptor ligands with *K*_i_ values of 0.52 and 0.16 nM, respectively [20].

Considering the pharmacological properties of arylpiperazine and indole moieties, our research group successfully designed and synthesized a series of indole-arylpiperazine derivatives. Biological evaluation showed that several compounds exhibit moderately good selectivity for α_1_-adrenoceptor subtypes [13]. However, the structure-activity relationship and the associated with binding mechanisms remain unclear, thereby hindering the design of novel α_1A_-adrenoceptor-selective antagonists. 

In this work, crystal structures, geometric parameters and intermolecular interactions of title compounds **1**, **2** and **3** were described. Energy gaps between the highest occupied molecular orbital (HOMO) and lowest unoccupied molecular orbital (LUMO) were obtained by theoretical calculations based on DFT-optimized conformations. Molecular electrostatic potential (MEP) was also used to indicate the reactive sites of electrophilic and nucleophilic attacks for these compounds. Molecular docking was then utilized to investigate the binding mechanisms of the ligands-α_1A_-receptor complexes. This work provides valuable information regarding the relationship of structure-activity-binding mechanism, and is particularly beneficial for pharmaceutical chemists.

## 2. Results and Discussion

### 2.1. Structural Characterizations

**Figure 2:**
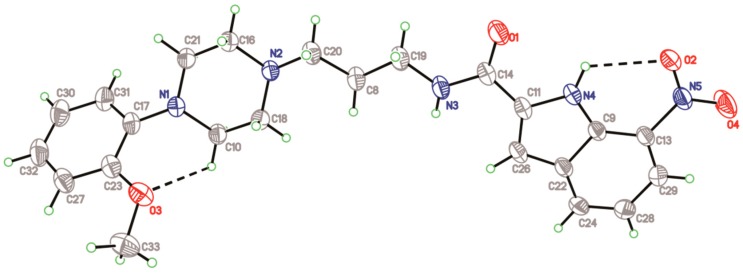
Crystallographic structure of compound **1**. Displacement ellipsoids are drawn at the 30% probability level, and intramolecular hydrogen bonds are presented in dashed lines.

Compound **1** crystallizes in the monoclinic space group *C2/c*. The atomic numbering and displacement ellipsoid plot are presented in Figure 2. Crystal data and structural refinement of compound **1** are presented in Table 1. Intramolecular H-bonds C(10)–H(10A)···O(3) and N(4)–H(4)···O(2) result in the formation of non-planar and planar six-membered pseudo rings, respectively. The dihedral angle formed between aromatic plane and indole ring is 44.20(6)°. The piperazine moiety displays a typical chair conformation, as the torsion angles of N(2)–C(18)–C(10)–N(1) and N(2)–C(16)–C(21)–N(1) are is −58.6(2)° and 58.7(2)°, respectively.

**Table 1:** Crystal data and structural refinement of compound **1**, **2** and **3**.

Empirical Formula	C_23_H_27_N_5_O_4_	C_24_H_30_N_4_O_3_	C_23_H_28_N_4_O_2_
Formula weight	437.49	422.52	392.49
Temperature (K)	293(2)	293(2)	293(2)
Crystal system	monoclinic	Triclinic	orthorhombic
Space group	*C2/c*	*P*-1	*P*ccn
a, b, c (Å)	21.385(4), 7.8960(16), 28.227(6)	9.998(2), 10.692(2), 13.614(3)	15.762(3), 35.112(7), 7.8452(16)
α, β, γ (°)	90, 93.13(3), 90	100.89(3), 105.05(3), 98.87(3)	90, 90, 90
Volume (Å^3^), *Z*	4759.2(17), 8	1348.0(5), 2	4341.9(15), 8
ρ_calc_ (g·cm^−3^)	1.221	1.041	1.201
μ/mm^−1^	0.7	0.561	0.624
F(000)	1856	452	1680
Crystal size (mm^3^)	0.3 × 0.2 × 0.2	0.3 × 0.2 × 0.2	0.3 × 0.25 × 0.23
θ range for data collection (°)	3.136–68.242	3.463–68.213	3.073–62.499
*h*, *k*, *l*	−25–25, −9–9, −33–33	−12–11, −12–12, −16–16	−18–18, −42–42, −8–9
Reflections collected	37,917	24,318	74,888
Independent reflections, R_int_	4353, 0.0731	4851, 0.0574	3455, 0.0485
Data/restraints/parameters	4353/0/290	4851/0/282	3455/85/294
Goodness-of-fit on *F*^2^	1.084	0.996	1.002
R_1_, *wR*_2_ [I ≥ σ (I)]	0.0550, 0.1426	0.0620, 0.1773	0.0592, 0.1474
R_1_, *wR*_2_ [all data]	0.0717, 0.1532	0.0806, 0.1935	0.0764, 0.1684
Largest diff. peak/hole (e·Å^−3^)	0.30/−0.21	0.38/−0.20	0.24/−0.18

**Figure 3:**
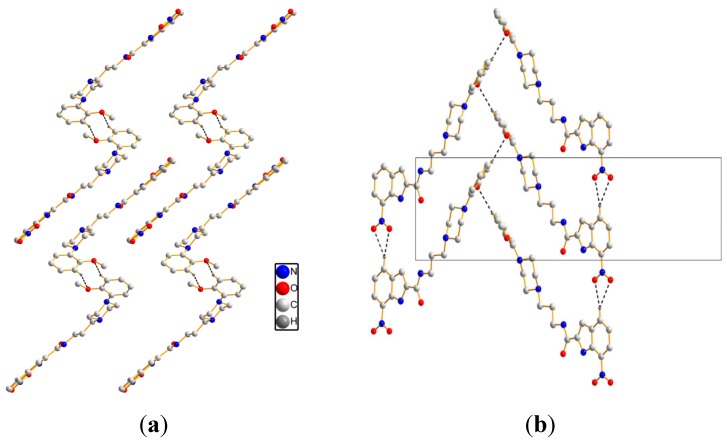
Crystal packing of **1**. (**a**) viewing along the *b* axis; (**b**) viewing along the *a* axis. Black dashed lines show the intermolecular hydrogen bonds.

As for crystal packing, two independent molecules form ${\text{R}}_{2}^{2}$(8) ring motif along the *b* axis, see Figure 3a. Simultaneously, intermolecular hydrogen bonds [C(24)–H(24)···O(2) and C(24)–H(24)···O(4)] are observed to realize a ${\text{R}}_{2}^{1}$ (4) ring motif along the *a* axis. The framework is further reinforced by zigzag H-bondings [C(27)–H(27)···O(3)], see Figure 3b. The detailed intermolecular H-bonds data are presented in Table 2.

**Table 2:** Intermolecular hydrogen bonds for compound **1** (Å, °).

D–H···A	D–H	H···A	D···A	D–H···A (°)
C(24)–H(24)···O(2) ^i^	0.93	2.49	3.318(3)	147.9(3)
C(24)–H(24)···O(4) ^i^	0.93	2.44	3.294(3)	152.4(2)
C(27)–H(27)···O(3) ^ii^	0.93	2.55	3.409(3)	154.4(3)

Symmetry code: (i) *x*, −1 + *y*, *z* (ii) 1/2 − *x*, −1/2 + *y*, 1/2 − *z*.

**Figure 4:**
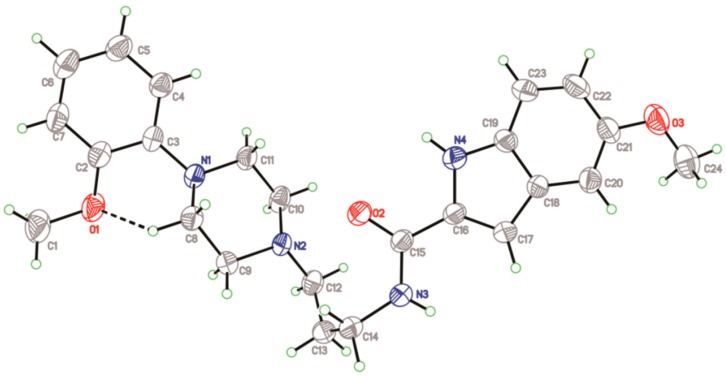
Crystallographic structure of compound **2**. Displacement ellipsoids are drawn at the 30% probability level, and intramolecular hydrogen bond is shown in dashed line.

The crystal of compound **2** gives triclinic form with space group *P*-1, see Figure 4. The crystal data and structural refinement of compound **2** are presented in Table 1. The dihedral angle of aromatic plane and indole ring is 11.2(9)°, which is obviously smaller in comparison to that of compound **1**. In order to explain the discrepancy of two specific conformations, we performed conformational analysis, see Table 3. As a comparison to the bond lengths, bond angles and torsion angles between **1** and **2**, significant differences are observed to be at the amide alkyl linkage, e.g., the torsion angle [N(2)–C(20)–C(8)–C(19)] of **1** is −179.0(2)° while corresponding angle [N(2)–C(12)–C(13)–C(14)] of **2** is −52.6(3)°. This case is most likely ascribed to the differences of intramolecular and intermolecular hydrogen bonds that promote the formation of specific conformers. 

**Table 3:** Selected geometric parameters of compound **1** and **2** (Å, °).

Compound 1		Compound 2	
Bonds	Dist.	Bonds	Dist.
N(1)–C(17)	1.419(2)	N(1)–C(3)	1.413(3)
N(1)–C(10)	1.464(2)	N(1)–C(8)	1.474(3)
N(3)–C(14)	1.337(2)	N(3)–C(15)	1.331(3)
N(4)–C(11)	1.393(2)	N(4)–C(16)	1.370(3)
C(8)–C(20)	1.512(3)	C(12)–C(13)	1.504 (4)
Angle	(°)	Angle	(°)
C(20)–C(8)–C(19)	110.64(17)	C(12)–C(15)–C(14)	115.9(2)
C(19)–N(3)–C(14)	122.09(18)	C(15)–N(3)–C(14)	121.7(2)
N(2)–C(20)–C(8)–C(19)	−179.0(2)	N(2)–C(12)–C(13)–C(14)	−52.6(3)
C(20)–C(8)–C(19)–N(3)	171.5(2)	C(12)–C(13)–C(14)–N(3)	−56.2(3)
C(8)–C(19)–N(3)–C(14)	171.2(2)	C(13)–C(14)–N(3)–C(15)	94.9(3)

**Figure 5:**
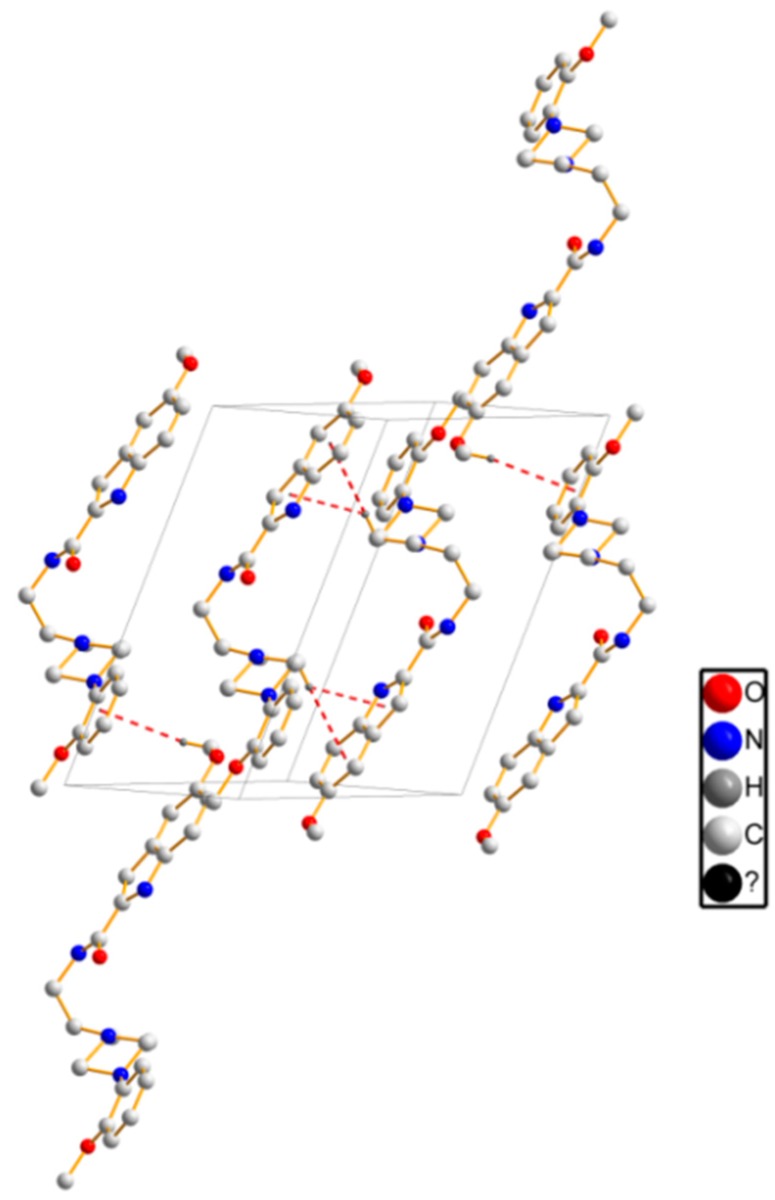
Crystal packing of **2**. Red dashed lines show the intermolecular C–H···π interactions.

Another particular interest for us in studying the crystal structure of **2** is to investigate the stability force comprising intermolecular interactions. As shown in Figure 5, C–H···π interactions are found to be the critical forces to stabilize the three-dimensional structure [21]. The geometry parameters of C–H···π interactions are listed in Table 4. Two molecules form a dimer of head-tail crosslinking through C(24)–H(24B)···π interactions. Additionally, a dimer constructed by two antiparallel molecules plays a significant role in stabilizing the packing structure. 

**Table 4:** C–H···π interaction geometry of compound **2** (Å, °).

···X–H	Cg *	H···Cg	X···Cg	X–H···Cg (°)
C(10)–H(10A) ^i^	N(4)–C(16)–C(17)–C(18)–C(19)	2.70	3.437(3)	133
C(10)–H(10A) ^i^	C(18)–C(19)–C(23)–C(22)–C(21)–C(20)	2.78	3.753(3)	178
C(24)–H(24B) ^ii^	C(2)–C(3)–C(4)–C(5)–C(6)–C(7)	3.00	3.917(4)	160

Symmetry code: (i) 1 − *x*, 1 − *y*, 1 – *z*; (ii) *x*, 1 + *y*, 1 + *z*. The centroids of the planar rings are presented by asterisk (*).

**Figure 6:**
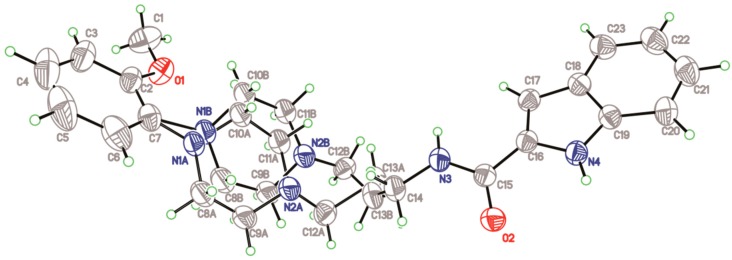
Crystallographic structure of compound **3**. Displacement ellipsoids are drawn at the 30% probability level.

**Figure 7:**
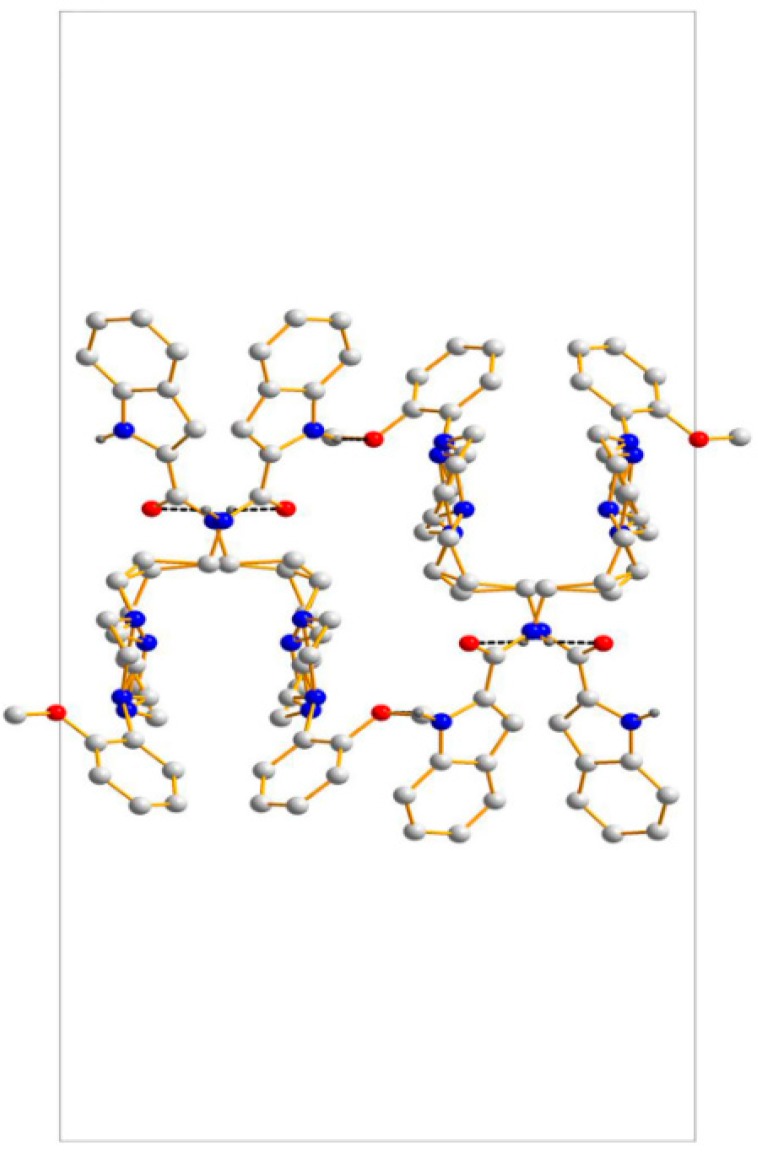
Crystal packing of **3** along the *c* axis. Black dashed lines show the intermolecular H-bonds.

Crystal structure of compound **3** was herein described clearly. It gives orthorhombic with space group *P*ccn. As shown in Figure 6, the piperazine ring, C(12) and C(13) atoms were disordered. The dihedral angle of aromatic plane and indole ring is 57.6(8)°. With respect to the packing structure, the intermolecular hydrogen bonds (Table 5) form network structure. The NH [N(3)–H(3), N(4)–H(4)] of a donor molecule realizes H-bond with the O atoms of an adjacent molecule, see Figure 7. Furthermore, compared to hydrogen bonds (3.294–3.409 Å) in compound **1**, the H-bonds (2.953–3.151 Å) in compound **3** have shorter bond lengths, indicating that higher bond energies contribute to the stability of the three-dimensional structure of **3**.

**Table 5:** Intermolecular hydrogen bonds for compound **3** (Å, °).

D–H···A	D–H	H···A	D···A	D–H···A (°)
N(3)–H(3)···O(2) ^i^	0.86	2.14	2.953 (4)	157.5 (2)
N(4)–H(4)···O(1) ^ii^	0.86	2.37	3.151 (1)	151.7 (2)

Symmetry code: (i) 1/2 − *x*, *y*, 1/2 + *z*; (ii) 1/2 + *x*, −*y*, 1/2 − *z*.

### 2.2. DFT Calculations

**Figure 8:**
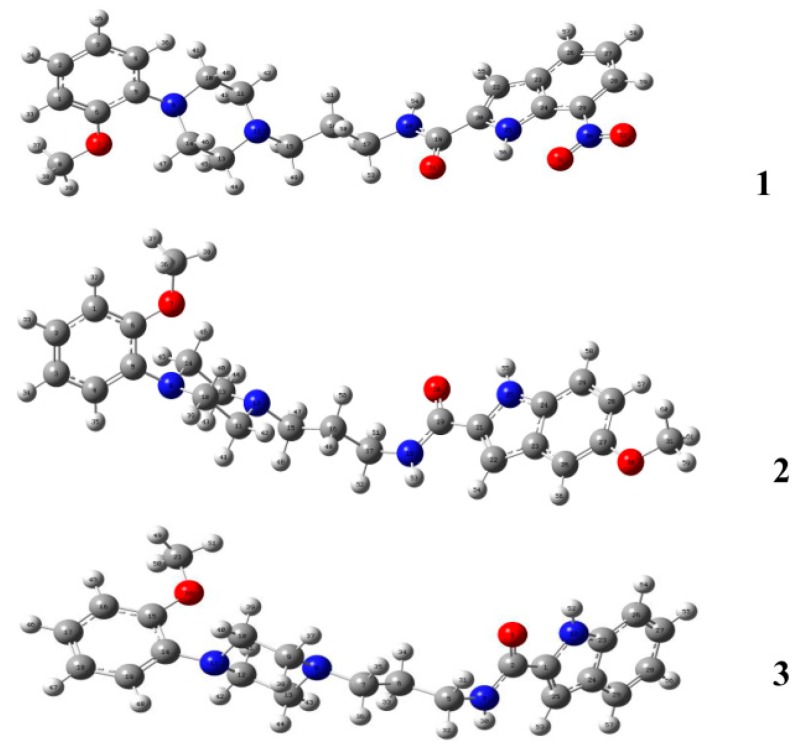
DFT-optimized conformations of compound **1**, **2** and **3** at the B3LYP/6-311G (d, p) level of theory.

Compounds **1**–**3** were subject to theoretical calculations using Gaussian 09 at the B3LYP/6-311G (d, p) level of theory to elucidate their structural properties. The optimized geometries are shown in Figure 8. The comparison of the calculated bond lengths and bond angles among compounds **1**–**3** and the corresponding solid-state values are listed in Table 6. The bond-length deviations between the calculated and solid-state conformers are 0.001 to 0.021 Å, and the deviations of bond angles range from 0.1 to 1.6°. The results suggest that there is no apparent difference between the theoretically calculated and experimental crystallographic conformations.

**Table 6:** Optimized geometrical parameters of theoretically calculated and solid-state conformers.

Compound 1		
Bond lengths (Å)	DFT	X-ray
O(7)–C(6)	1.362	1.373(3)
N(25)–C(24)	1.354	1.362(2)
C(15)–C(16)	1.525	1.511(3)
C(27)–C(28)	1.396	1.385(3)
Bond angles (°)		
C(20)–C(19)–N(18)	116.5	115.8(2)
C(15)–C(16)–C(17)	111.5	110.8(2)
N(12)–C(15)–C(16)	113.3	114.9(2)
**Compound 2**		
Bond lengths (Å)	DFT	X-ray
O(7)–C(6)	1.36	1.380(3)
N(25)–C(24)	1.371	1.370(3)
C(15)–C(16)	1.527	1.515(4)
C(27)–C(28)	1.409	1.394(4)
Bond angles (°)		
O(21)–C(19)–C(18)	123.4	122.6(2)
C(15)–C(16)–C(17)	111.5	115.9(2)
N(12)–C(15)–C(16)	113.5	114.5(2)
**Compound 3**		
Bond lengths (Å)	DFT	X-ray
O(4)–C(2)	1.221	1.242(3)
N(22)–C(23)	1.366	1.371(3)
C(5)–C(7)	1.524	1.506(9)
C(14)–C(19)	1.385	1.376(4)
Bond angles (°)		
O(4)–C(2)–N(3)	123.4	122.3(2)
O(20)–C(15)–C(14)	116.0	115.0(2)
N(22)–C(23)–C(24)	107.4	107.5(2)

**Figure 9:**
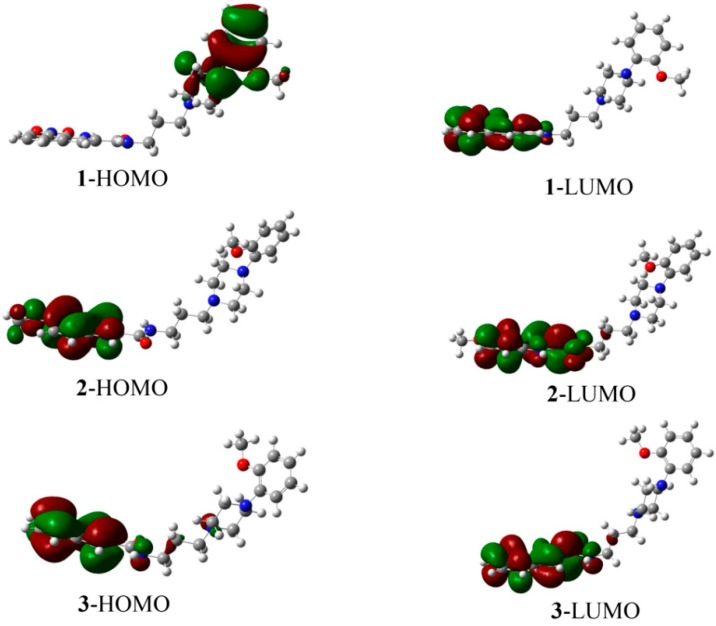
HOMO, LUMO surfaces of compound **1**, **2** and **3** simulated at the B3LYP/6-311G (d, p) level of theory.

Molecular orbitals are very useful for physicists and chemists because the energy gap between HOMO and LUMO characterizes the chemical reactivity and kinetic stability of molecule [22]. As shown in Figure 9, the HOMO of **1** is localized on the arylpiperazine moiety, whereas the LUMO is mainly concentrated on the 7-nitro-1*H*-indole moiety. The HOMO and LUMO orbitals of **2** and **3** are localizes over the amide and indole moieties. The HOMO-LUMO energy gaps are 2.728, 4.324 and 4.610 eV for compound **1**, **2** and **3**, respectively (Table 7).

**Table 7:** Frontier molecular orbital energies of **1**, **2** and **3** (eV).

Compound	*E*_HOMO_	*E*_LUMO_	*∆E*_LUMO–HOMO_
**1**	−5.395	−2.667	2.728
**2**	−5.508	−1.184	4.324
**3**	−5.807	−1.197	4.610

Chemical hardness is a useful concept for understanding the behavior of chemical systems and is associated with the stability and reactivity of a chemical system. The chemical hardness (ƞ) can be expressed as: ƞ = (–*E*_HOMO_ + *E*_LUMO_)/2. Using HOMO and LUMO orbital energies, we determined that the values of ƞ**_1_**, ƞ**_2_** and ƞ**_3_** are 1.364, 2.162 and 2.305 eV, respectively. The results suggest that **1** is more reactive and less stable than **2** and **3**. Electronic chemical potential (µ) is defined as the escaping tendency of electrons from an equilibrium system and is given by µ= (*E*_HOMO_ + *E*_LUMO_)/2. The µ values increase in the order of **1** (–4.031 eV) < **3** (–3.502 eV) < **2** (–3.346 eV). Generally, hard molecules will have their electron density changed more hardly than a soft molecule [23]. In this case, the chemical hardness of **3** is larger than that of **2**, but the electronic chemical potential of the former is slightly smaller than the latter. It may be ascribed to the intermolecular hydrogen bonds of **3** that facilitate the electronic transfers. The global electrophilicity power of a ligand, expressed as ω = µ^2^/2ƞ, is a measure of the stabilization in energy achieved when the system acquires an additional electronic charge from the environment. The corresponding ω values of **1**, **2** and **3** are 5.956, 2.589 and 2.660, respectively.

**Figure 10:**
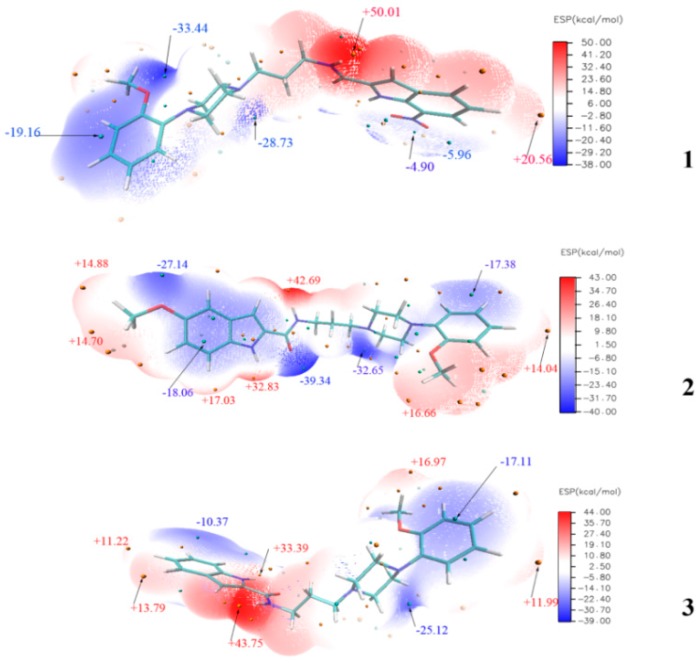
MEP surfaces of compound **1**, **2** and **3**.

**Figure 11:**
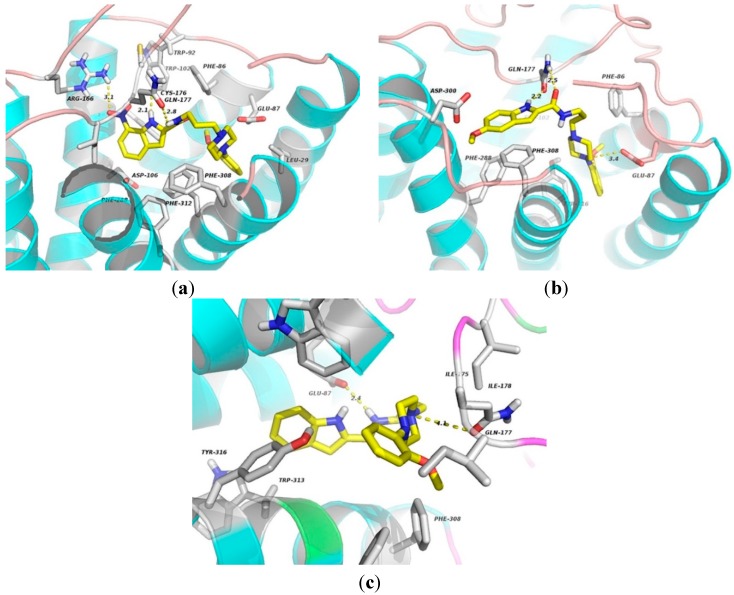
The docking complexes of ligands **1** (**a**); **2** (**b**); and 3 (**c**) with α_1A_-AR. These compounds are shown in stick representation. The elements are coloured as follows: oxygen, red; nitrogen, blue; carbon, yellow. Dashed lines represent the hydrogen bonds or electrostatic interactions.

The molecular electrostatic potential (MEP) maps were evaluated using the B3LYP method with the basis set of 6-311G (d, p) to investigate the reactive sites of electrophilic and nucleophilic attacks for the title compounds. The positive (red) regions of MEP were related to nucleophilic reactivity, and the negative (blue) regions referred to electrophilic reactivity. Determining electrostatic potential is a suitable process for analysis based on “recognition” of one molecule by another, similar to that in drug receptor and enzyme substrate interactions, because the two species detect each other through their potentials [24,25]. As shown by the MEP map of **1** (Figure 10), the negative regions are mainly localized over the anisole ring. The maximum positive region is over the NH portion of the amide group (+50.01 kcal/mol). Similar to that of compound **1**, the maximum positive region of **2** is localized to its own NH group (+42.69 kcal/mol), and the maximum negative site is concentrated on the O atom of the amide group (–39.34 kcal/mol). The N atom in the piperazine ring (–32.65 kcal/mol) also displays significant electrophilic reactivity. Nevertheless, the NH of indole ring exhibits a strong nucleophilic reactivity (+32.83 kcal/mol). These findings suggest that the above-mentioned sites are involved in the hydrogen-bonding interactions, which is validated by molecular model of the **2**-α_1A_ complex (Figure 11b). The maximum positive region for **3** is also localized to the NH group (+43.75 kcal/mol).

### 2.3. Binding Mechanisms of the Ligand-α_1A_ Complex

G protein-coupled receptors (GPCRs) are the largest class of membrane receptors in eukaryotes, and are characterized by seven transmembrane (TM) helices, and N- and C-terminal fragments. The TM helices are connected by alternating intra- and extracellular loop (ECL) regions that are very flexible and important for a wide range of biological functions. When agonists or antagonists bind to GPCRs, the hosts act as molecular switches that modulate downstream effector proteins when turned on [26]. α_1A_-Adrenoceptor is a member of the GPCR family. The accurate crystal structure of α_1A_ at the atomic resolution cannot be obtained, thereby severely impeding the development of therapeutic BPH medicines through structure-based drug design. Nevertheless, homology-modeling procedures with continuous progresses provide viable tools for structure-based GPCR ligand design.

Functional experiments involving animal tissues validated that compound **1**, **2** and **3** exhibit 11.0-, 18.6- and 4.7-fold increases in selectivity, respectively, for α_1A_ over than the α_1B_ subtype [13]. The minimum-energy structures of the ligands were subject to molecular docking using a homology model of the α_1A_ receptor to determine the structure-activity-binding mechanism relationship. As shown in Figure 10a, NH in the indole ring as a donor forms H-bond (2.1 Å) with Cys176 residue. The NH of amino group realizes a hydrogen bond (2.8 Å) with the carbonyl oxygen atom of Gln177 in the ECL2 region that has been proved to be essential for GPCR activation [27]. The indole and benzene rings mainly engage in hydrophobic interactions with Trp102, Trp92, Phe86, Phe308, Phe288 and Phe312. The residues including Asp106, Glu87 and Leu29 participate in **1** binding to the α_1A_ receptor with van der Waals interactions. In the **2**-α_1A_-adrenoceptor complex, the NH in indole ring and the carbonyl oxygen atom are involved in two hydrogen bonds with the nitrogen and oxygen atoms of Gln177 (2.2 and 2.5 Å). In addition, **2** contacts via hydrophobic interactions with residues Phe86, Tyr316 and Phe288. The indole ring forms aromatic π-stacking against Phe308 of TM7 (Figure 10b). The cationic nitrogen atom in the piperazine ring for the electrostatic binding to Glu87 of TM2 is also presented. As observed from **3**-α_1A_ complex (Figure 10c), the NH of amino group can realize a H-bond (2.4 Å) with Glu87 of ECL1. The indole and benzene rings for hydrophobic interactions with the region formed by Tyr316, Trp313, Phe308 of TM7 and Phe288 of TM6 are found. The arylpiperazine moiety is in weak van der waals contact with residues Ile175 and Ile178 of ECL2. The protonated piperazine moiety forms a weak electrostatic interaction with Gln177 (4.1 Å between the nitrogen atom of piperazine ring and the amide oxygen atom of Gln177).

**Figure 12:**
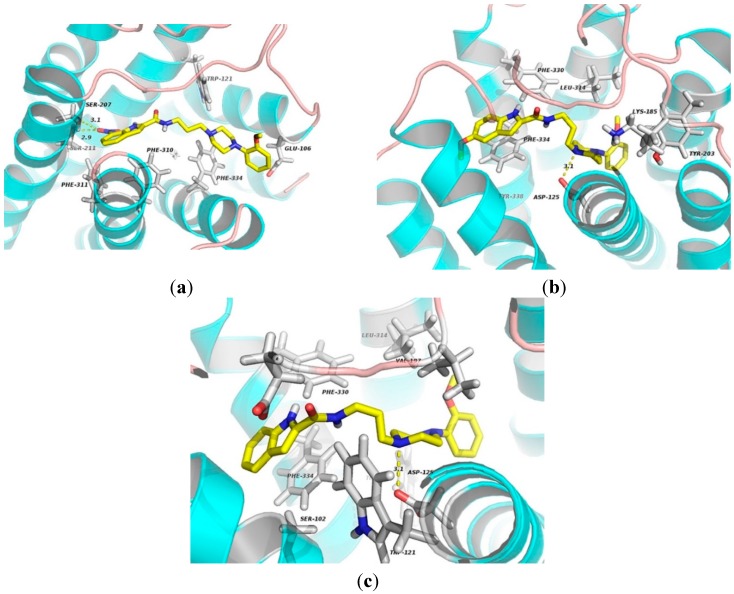
The docking complexes of ligands **1** (**a**); **2** (**b**); and **3** (**c**) with α_1B_-AR. These compounds are shown in stick representation. The elements are coloured as follows: oxygen, red; nitrogen, blue; carbon, yellow. Dashed lines represent the hydrogen bonds or electrostatic interactions.

To shed some light on the selectivity for α_1A_ over than α_1B_ subtype, molecular dockings using a homology of α_1B_ receptor were performed. The results show that the oxygen atom of nitrate group of **1** forms two hydrogen bonds with residues Ser207 and Ser211 (3.1 and 2.9 Å) of TM5. The indole and benzene rings mainly engage in hydrophobic interactions with Phe310, Phe311, Phe334 and Trp121 residues (Figure 12a). As for **2**-α_1B_ complex, the ligand contacts via hydrophobic interactions with Phe330, Phe334 and Tyr203. Other residues, including Leu134 and Lys185, involve in ligand binding by van der Waal’s forces. Additionally, the cationic nitrogen atom in the piperazine moiety for electrostatic binding to Asp125 (3.1 Å) is also observed as shown in Figure 11b. One residue Phe334 in TM7 may be an important binding site for **3**-α_1A_ complex by π-stacking interaction. Compound **3** contacts via van der Waals interactions with residues Ser102, Glu194, Val197 and Leu134. The indole moiety involves in hydrophobic interactions with Phe330 and Trp307. In addition, the electronegative atom oxygen in Asp125 of TM3 makes an electrostatic interaction with the nitrogen atom of the piperazine ring.

Studies on the binding mechanism indicate that residues Gln177, Phe86, Phe308, Phe288 and Glu87 are identified as the major sites for indole-arylpiperazine derivatives (**1**, **2** and **3**) binding to α_1A_ receptor. On the other hand, the important sites for ligands binding with α_1B_ subtype are involved in Asp125, Leu134, Phe334, Phe330, Ser207 and Ser211 residues. We also noticed that the Gln177 residue in ECL2 seems to play a significant role in improving the selectivity of α_1A_ against α_1B_ subtype. The ligands binding to residue Asp125 by electrostatic interactions and contacting Phe334 via hydrophobic interactions or π-stacking interactions contribute to increased affinity for α_1B_ receptor.

## 3. Experimental Section

### 3.1. Chemical Synthesis

The title compound **1**, **2** and **3** were synthesized by our previous work [13], and characterized by ^1^H- and ^13^C-NMR.

### 3.2. X-ray Crystallography 

Suitable crystals of compound **1**–**3** were obtained by slowly evaporating a mixture of dichloromethane and *n*-hexane solution at ambient temperature, and single crystal of compound **2** was obtained from a solution of tetrahydrofuran and *n*-pentane. High-quality colorless crystals were mounted on a glass fiber in a random orientation. The data were collected by a R-AXIS RAPID II diffractometer (Rigaku Co., Ltd., Matsubaracho Akishima, Japan) equipped with graphite-monochromatic Cu-*K*α radiation (λ = 1.54178 Å) by using a ω scan mode. The structures were solved by direct methods using Olex2 software [26], and the non-hydrogen atoms were located from the trial structure and then refined anisotropically with SHELXL using a full-matrix least squares procedure based on *F*^2^ [28]. The weighted *R* factor, *wR* and goodness-of-fit *S* values were obtained based on *F*^2^. The hydrogen atom positions were fixed geometrically at the calculated distances and allowed to ride on the parent atoms. CCDC 1007945 (**1**), 1009714 (**2**), and 1021886 (**3**) contain the supplementary crystallographic data for this paper. These data can be obtained free of charge via http://www.ccdc.cam.ac.uk/conts/retrieving.html (or from the CCDC, 12 Union Road, Cambridge CB2 1EZ, UK; Fax: +44 1223 336033; E-mail: deposit@ccdc.cam.ac.uk.

### 3.3. Theoretical Calculations

The initial conformational optimizations were performed at the molecular mechanics level of theory employing MM+ force fields incorporated in HyperChem 7.5 software package [29]. The conformer further submitted to the Gaussian 09 for DFT optimization at the B3LYP/6-311G (d, p) level of theory. Harmonic vibration frequencies were calculated to confirm the stability of these conformers [30]. As revealed by the frequency analysis, no imaginary frequencies were observed in ground states. Molecular orbitals were plotted by using GaussView (version 5.0.8, Gaussian Inc., Wallingford, CT, USA). MEP maps were constructed by the Multiwfn program [31].

### 3.4. Homology Modeling and Molecular Docking

The amino acidic sequence of the human α_1A_ receptor was retrieved from SwissProt database (entry code P35348, A1AA_HUMAN) [32]. Based on Zhou’s work [33], the 3D model of α_1A_ was perfectly achieved with a TM-score of 0.65. Generally, models with TM-score > 0.4 have significant similarity to native structures. The model was then submitted to be energy optimization by using CHARMMing program [34]. The loop conformations of energy-optimized model were reasonably modified by the Scwrl4 software [35], which produced the final homology model of α_1A_ receptor. Stable conformations of **1**, **2** and **3** that were extracted from the single-crystal structures were saved in PDB format. Autodock vina was employed to perform the molecular docking [36,37], and the preferential binding mode was finally selected according to the lowest affinity energy (kcal/mol). Afterward, the full color was obtained by using the program PyMOL. The human α_1B_ model was obtained according to the reported literature [38]. 

## 4. Conclusions

Numerous indole-arylpiperazine derivatives exhibit considerable selectivity for α_1_-AR subtype, but the structure-activity-binding mechanism relationship remains unclear. In the present work, compounds **1**, **2** and **3** were investigated using single-crystal X-ray diffraction analysis, theoretical calculations and molecular docking based on the homology models of α_1A_ and α_1B_ receptors. Crystals of **1**, **2** and 3 belong to the monoclinic form with *C2/c* space group, triclinic form with space group *P*-1 and orthorhombic with space group *P*ccn, respectively. Intra- and intermolecular hydrogen bonds play a significant role in the packing structures of **1**. For compound **2**, C–H···π interactions are critical forces to stabilize the three-dimensional structure. The intermolecular H-bonds of **3** form a network structure, and have higher bonds energies by comparison to that of **1**. The HOMO-LUMO energy gaps were obtained by DFT calculations at B3LYP/6-311G (d, p) level of theory. The results suggest that compound **1** is less kinetically stable than compound **2** and **3**. MEP maps of these compounds indicate that the positive regions are mainly localized over the NH group, and the regions of electrophilic reactivity are distributed in the C=O group and N atom in the piperazine ring. Molecular docking of ligand-α_1_-receptor complex was performed to illustrate the structure-activity-binding mechanism relationship. Residues Gln177, Phe86, Phe308, Phe288 and Glu87 are identified as the major sites for ligands binding to α_1A_ receptor while the important sites for ligands binding with α_1B_ subtype are involved in Asp125, Leu134, Phe334, Phe330, Ser207 and Ser211 residues. The present work provides valuable clues for designing α_1A_-selective antagonists with better efficacy.

## Supplementary Materials

Supplementary materials can be accessed at: http://www.mdpi.com/1420-3049/20/11/19651/s1.
