# Supplementary material for: X-ray Crystallography, DFT Calculations and Molecular Docking of Indole-Arylpiperazine Derivatives as α1A-Adrenoceptor Antagonists
Source: Molecules. 2015 Oct 30;20(11):19674–89. doi: 10.3390/molecules201119651 (PMC6332402; doi:10.3390/molecules201119651)
Supplement: Supplementary file 1 [file molecules-20-19651-s001.pdf]

molecules

Supplementary Informations

Table S1. Cartesian coordinates of DFT-optimized conformation for compound **1**.

Tag	Symbol	X	Y	Z
1	C	−8.7773726	1.0422148	0.2743532
2	C	−8.8054903	2.3930464	−0.0639906
3	C	−7.6394743	3.0326383	−0.4274994
4	C	−6.4396314	2.3268731	−0.4398698
5	C	−6.3802336	0.9777684	−0.1107024
6	C	−7.5879136	0.3327595	0.2429994
7	O	−7.495917	−0.9958697	0.5278195
8	C	−8.6812501	−1.7066036	0.8133193
9	N	−5.1876371	0.230985	−0.1669911
10	C	−4.0885342	0.7813729	−0.93748
11	C	−3.0499281	−0.2982681	−1.1971673
12	N	−2.5587226	−0.8579853	0.0526315
13	C	−3.6685827	−1.4032546	0.8217315
14	C	−4.7113685	−0.3352567	1.0933237
15	C	−1.4986048	−1.8327829	−0.1423144
16	C	−0.1600266	−1.2021627	−0.5091792
17	C	0.9404572	−2.2530608	−0.6276626
18	N	2.2378673	−1.6887148	−0.958454
19	C	3.103951	−1.2955068	0.0040293
20	C	4.3795047	−0.6943601	−0.4588353
21	O	2.8907028	−1.4203488	1.1970861
22	C	4.9395746	−0.485528	−1.6877757
23	C	6.2074852	0.1363902	−1.4636866
24	C	6.3462883	0.2699642	−0.0582959
25	N	5.2307262	−0.2381431	0.5175015
26	C	7.2275224	0.5851206	−2.3053015
27	C	8.3609268	1.1554015	−1.7582194
28	C	8.5002906	1.2883939	−0.3745573
29	C	7.5040789	0.8508038	0.4723297
30	N	7.6646248	0.9952453	1.9116193
31	O	6.7511962	0.5879514	2.6152092
32	O	8.68084	1.5045548	2.3349252
33	H	−9.6986751	0.5459434	0.5446313
34	H	−9.7464824	2.9287599	−0.0428041
35	H	−7.6468964	4.0824426	−0.6935851
36	H	−5.528319	2.8437064	−0.7088331
37	H	−9.3864125	−1.6601629	−0.0225156
38	H	−9.1693704	−1.3315528	1.7186395
39	H	−8.3806915	−2.7402912	0.9708532
40	H	−3.5983081	1.6221573	−0.4189311
41	H	−4.471379	1.1470122	−1.8918357
42	H	−2.2186622	0.1437566	−1.7490655
43	H	−3.5012475	−1.0822047	−1.8324067

Table S1. *Cont.*

Tag	Symbol	X	Y	Z
44	H	−3.2830111	−1.7782888	1.7728358
45	H	−4.1531595	−2.2436839	0.2933733
46	H	−4.2659833	0.4514566	1.7228142
47	H	−5.550131	−0.7753788	1.6254538
48	H	−1.7829216	−2.5856306	−0.9022898
49	H	−1.3739284	−2.3752733	0.799176
50	H	0.1124666	−0.4800322	0.2631507
51	H	−0.2342045	−0.655606	−1.4537836
52	H	0.6875967	−2.9943724	−1.3900039
53	H	1.0577425	−2.7774112	0.3211783
54	H	2.4438195	−1.4681713	−1.9181315
55	H	4.5220203	−0.7458486	−2.6473866
56	H	5.0442398	−0.2923583	1.5083249
57	H	7.1296904	0.4863727	−3.3799396
58	H	9.1561745	1.5061005	−2.4028126
59	H	9.3834317	1.7327662	0.0621286

Table S2. Cartesian coordinates of DFT-optimized conformation for compound **2**.

Tag	Symbol	X	Y	Z
1	C	8.00274	2.188816	0.266158
2	C	9.048073	1.694813	−0.515312
3	C	8.886506	0.527525	−1.249438
4	C	7.665648	−0.146058	−1.197116
5	C	6.602513	0.319478	−0.427779
6	C	6.78292	1.509924	0.314961
7	O	5.711401	1.924175	1.054027
8	C	5.826857	3.115835	1.818615
9	N	5.38271	−0.431316	−0.426308
10	C	4.22305	0.235256	−1.027295
11	C	3.100813	−0.778958	−1.233767
12	N	2.737061	−1.412728	0.034622
13	C	3.905236	−2.076014	0.615558
14	C	5.033755	−1.074626	0.845254
15	C	1.596141	−2.317299	−0.081802
16	C	0.258026	−1.59105	−0.251166
17	C	−0.923275	−2.569995	−0.238892
18	N	−2.21365	−1.918829	−0.420006
19	C	−3.034974	−1.62113	0.624402
20	O	−2.783698	−1.916769	1.789173
21	C	−4.29361	−0.912982	0.292062
22	C	−4.870679	−0.478818	−0.883401
23	C	−6.110879	0.14483	−0.538214
24	C	−6.233412	0.0559	0.879042

Table S2. *Cont.*

Tag	Symbol	X	Y	Z
25	N	−5.114776	−0.589653	1.347345
26	C	−7.125375	0.765414	−1.279945
27	C	−8.229275	1.280539	−0.613509
28	C	−8.336649	1.184095	0.792814
29	C	−7.342877	0.573439	1.545918
30	O	−9.17895	1.873189	−1.406402
31	C	−10.329594	2.429369	−0.793621
32	H	8.147186	3.099428	0.831929
33	H	9.988666	2.233638	−0.542293
34	H	9.696642	0.141865	−1.857001
35	H	7.506111	−1.059524	−1.758144
36	H	6.027527	3.983965	1.18118
37	H	6.613165	3.032208	2.576851
38	H	4.864827	3.246048	2.311431
39	H	4.522965	0.644915	−1.995396
40	H	3.851906	1.061077	−0.403358
41	H	3.424322	−1.533003	−1.976945
42	H	2.23052	−0.258938	−1.640845
43	H	4.273524	−2.892069	−0.034678
44	H	3.614804	−2.515026	1.574709
45	H	5.915238	−1.60078	1.220656
46	H	4.71826	−0.344061	1.604184
47	H	1.551649	−2.90602	0.840485
48	H	1.737372	−3.042077	−0.908553
49	H	0.24207	−1.024843	−1.188509
50	H	0.141342	−0.870427	0.562693
51	H	−0.971257	−3.089483	0.719149
52	H	−0.803068	−3.324561	−1.023479
53	H	−2.460686	−1.582419	−1.336773
54	H	−4.472008	−0.59128	−1.880118
55	H	−4.873456	−0.830284	2.296533
56	H	−7.074092	0.855575	−2.358187
57	H	−9.200729	1.588268	1.301839
58	H	−7.436251	0.505715	2.623731
59	H	−10.927338	2.843156	−1.604722
60	H	−10.069901	3.232974	−0.094287
61	H	−10.918817	1.669062	−0.267185

Table S3. Cartesian coordinates of DFT-optimized conformation for compound **3**.

Tag	Symbol	X	Y	Z
1	C	−5.11135	−0.398594	−0.151955
2	C	−3.903168	−1.083161	−0.668603
3	N	−3.058298	−1.605794	0.262289
4	O	−3.712162	−1.164201	−1.878549
5	C	−1.814192	−2.273862	−0.095126
6	C	−0.578379	−1.375579	0.048038
7	C	0.710118	−2.137579	−0.276916
8	N	1.904398	−1.296126	−0.279329
9	C	3.022677	−1.949342	−0.962381
10	C	4.223601	−1.012537	−1.053263
11	N	4.616562	−0.591732	0.29589
12	C	3.509353	0.068263	0.994633
13	C	2.312358	−0.876754	1.062763
14	C	5.891609	0.052227	0.400209
15	C	6.170227	1.319167	−0.164437
16	C	7.439129	1.885731	−0.022558
17	C	8.436494	1.203554	0.67555
18	C	8.178527	−0.04072	1.234754
19	C	6.908965	−0.601387	1.090602
20	O	5.140292	1.917952	−0.832624
21	C	5.355444	3.193405	−1.419908
22	N	−5.956338	0.15207	−1.090979
23	C	−7.022864	0.742154	−0.465236
24	C	−6.845199	0.557258	0.936636
25	C	−5.625638	−0.169714	1.106416
26	C	−8.127711	1.422404	−0.989368
27	C	−9.058176	1.9185	−0.091482
28	C	−8.901877	1.746329	1.300358
29	C	−7.810178	1.073841	1.819712
30	H	−3.25683	−1.444969	1.236631
31	H	−1.924294	−2.600691	−1.129979
32	H	−1.710022	−3.16749	0.529303
33	H	−0.544592	−0.977921	1.067826
34	H	−0.668226	−0.52181	−0.628879
35	H	0.612196	−2.565494	−1.280236
36	H	0.822565	−2.993407	0.418672
37	H	2.701621	−2.22117	−1.97243
38	H	3.328132	−2.876626	−0.441564
39	H	3.967625	−0.156413	−1.694373
40	H	5.063461	−1.543263	−1.509047
41	H	3.205374	1.001025	0.497888
42	H	3.835776	0.311225	2.009189
43	H	1.482894	−0.354375	1.545011
44	H	2.574065	−1.750806	1.689896
45	H	7.659999	2.854271	−0.451075

Table S3. *Cont.*

Tag	Symbol	X	Y	Z
46	H	9.416139	1.657413	0.776136
47	H	8.951332	−0.572267	1.77734
48	H	6.673619	−1.57069	1.514496
49	H	6.142134	3.157233	−2.181404
50	H	5.614352	3.944305	−0.665378
51	H	4.412098	3.46624	−1.889948
52	H	−5.761451	0.084965	−2.078588
53	H	−5.197761	−0.482382	2.046886
54	H	−8.250783	1.556102	−2.05811
55	H	−9.925851	2.450367	−0.464871
56	H	−9.653283	2.149854	1.969381
57	H	−7.698206	0.946206	2.890873
